# Fish Health Enhancement and Intestinal Microbiota Benefits of Asian Seabass (*Lates calcarifer* Bloch, 1790) on Dietary Sea Lettuce (*Ulva rigida* C. Agardh, 1823) Extract Supplementation

**DOI:** 10.3390/ani15121714

**Published:** 2025-06-10

**Authors:** Nawanith Klongklaew, Sanikan Tansutaphanit, Pornphimon Tiewpair, Wararut Buncharoen, Jitraporn Phaksopa, Prapansak Srisapoome, Anurak Uchuwittayakul

**Affiliations:** 1 Technical Group, Coastal Aquaculture Research and Development Division, Department of Fisheries, Bangkok 10900, Thailand; nawanith@gmail.com; 2 Chachoengsao Coastal Aquaculture Research and Development Center, Coastal Aquaculture Research and Development Division, Department of Fisheries, Chachoengsao 24130, Thailand; sanikarn@gmail.com; 3 Surat Thani Coastal Aquaculture Research and Development Center, Coastal Aquaculture Research and Development Division, Department of Fisheries, Surat Thani 84160, Thailand; pornphimontew@gmail.com; 4 Department of Biology, Faculty of Science, Chiang Mai University, Chiang Mai 50200, Thailand; wararut.bun@cmu.ac.th; 5 Department of Marine Science, Faculty of Fisheries, Kasetsart University, Bangkok 10900, Thailand; ffisjpp@ku.ac.th; 6 Laboratory of Aquatic Animal Health Management, Department of Aquaculture, Faculty of Fisheries, Kasetsart University, Bangkok 10900, Thailand; ffispssp@ku.ac.th; 7 Center of Excellence in Aquatic Animal Health Management, Faculty of Fisheries, Kasetsart University, Bangkok 10900, Thailand

**Keywords:** sea lettuce, *Ulva rigida*, Asian seabass, *Lates calcarifer*, antioxidant, immune responses, *Vibrio vulnificus*, sulfate polysaccharides

## Abstract

This study investigates the health benefits of supplementing Asian seabass diets with hot water crude extract from the sea lettuce *Ulva rigida* (*Ur*-HWCE). The extract’s proximate composition consists of 57.63% carbohydrates, 6.75% protein, 31.96% ash, and 6.01% sulfate polysaccharides, as confirmed by FTIR spectrum analysis. It also exhibits significant antioxidant properties, including total antioxidants, ABTS, DPPH, and reducing power. The study involved four groups fed *Ur*-HWCE at 0.5, 1.0, and 5 g/kg compared to a control group, with feed prepared daily and given twice at 5% of body weight for 4 weeks. *Ur*-HWCE supplementation did not negatively impact growth performance. It significantly upregulated insulin-like growth factor 1 (*igf1*) in the brain and liver, enhancing growth processes. *Ur*-HWCE reduced oxidative stress markers, such as malondialdehyde (MDA). Enhanced immune responses were observed, including increased bactericidal activity, serum IgM levels, and the upregulation of immune-related genes (*dcs*, *c3*, *ighm*, *lyz*, *il8*, *il10*). Gut microbiota analyses showed increased beneficial aerobic and natural probiotic *Bacillus* spp., particularly *Bacillus amyloliquefaciens*, enhancing gut health by reducing pathogenic bacteria. Blood biochemical parameters remained stable, and no histopathological alterations were found in the liver and intestine tissues, confirming the supplement’s safety. Fish fed with *Ur*-HWCE showed significantly higher survival rates and relative percent survival (RPS) against *Vibrio vulnificus* AAHM-VV2312 compared to the control group, demonstrating improved disease resistance. The study concludes that *Ur*-HWCE is a promising dietary supplement for enhancing the health, growth, and disease resistance of Asian seabass, supporting its potential in sustainable aquaculture practices.

## Simple Summary

This study tested a seaweed extract (*Ur*-HWCE) made from *Ulva rigida* (sea lettuce) as a supplement in the diet of Asian seabass. The extract is rich in carbohydrates, minerals, and antioxidants. Fish were fed different levels of the extract for 4 weeks. The supplement did not harm fish growth—in fact, it helped by boosting growth genes and reducing stress. It also improved the immune system, increased good gut bacteria (especially *Bacillus amyloliquefaciens*), and helped protect the fish from infection by *Vibrio vulnificus*. No side effects were found in the blood or organs. Overall, *Ur*-HWCE is a safe and effective supplement to improve the health and disease resistance of Asian seabass.

## 1. Introduction

The aquaculture industry has seen significant growth in recent years, driven by the increasing global demand for seafood. Among the various species farmed, Asian seabass (*Lates calcarifer* Bloch, 1790), also known as barramundi, is highly valued for its rapid growth, adaptability to diverse environments, and high market demand [1,2]. In 2022, Thailand produced 45,000 tons of Asian seabass, with a market value exceeding 150 million USD (FAO, 2022) [3]. The extensive farming of Asian seabass, particularly in high-density commercial aquaculture systems, has introduced challenges related to water quality maintenance and frequent disease outbreaks. However, the intensive farming practices required to meet this demand pose several challenges, including disease outbreaks, oxidative stress, and compromised fish immunity and growth performance [4]. One of the significant diseases affecting Asian seabass is caused by *Vibrio* species. These species include *Vibrio vulnificus*, *Vibrio harveyi*, *Vibrio alginolyticus*, and *Photobacterium damselae* (previously known as *Vibrio damsela*) [5,6,7,8]. Each pathogen capable of causing disease in marine fish poses a significant threat to aquaculture, leading to substantial economic losses and management challenges, particularly affecting the cultivation and post-transportation stages of Asian seabass.

Macroalgae, commonly known as seaweeds, are foundational to marine ecosystems due to their rapid growth rates and their integral role in biogeochemical cycles, where they mitigate environmental impacts by sequestering CO_2_, nitrogen, and phosphorus. Beyond their ecological contributions, macroalgae offer substantial benefits in aquaculture, serving as sustainable aquafeed ingredients because of their rich nutritional profile, which includes proteins, essential amino acids, and a notable content of omega-3 and omega-6 fatty acids [9]. These seaweeds are also replete with a spectrum of bioactive nutrients, such as vitamins, minerals, pigments, and antioxidants, which play a crucial role in the health and strength of aquaculture species [10].

Despite these advantages, the application of macroalgae in aquafeeds is often limited by their high content of structural and storage polysaccharides, such as cellulose, xylans, and laminarin, which can pose digestive challenges for fish [11]. Green macroalgae, particularly the *Ulva* species, especially *Ulva rigida*, known for its propensity to form sea lettuce in nutrient-rich waters, contain ulvan, a sulfated polysaccharide with a variety of biological activities [12]. Recent advancements have explored the potential of natural supplements derived from the hot water extracts of *U. rigida*. These extracts are rich in essential nutrients and possess antimicrobial, anti-inflammatory, and immune-enhancing properties [13]. Notably, such extracts have demonstrated potential in combating pathogenic *Vibrio* species. Remarkably, at 5 and 10 mg/mL, this extract could inhibit the growth of white spot syndrome virus (WSSV) in shrimp, evidenced by reduced viral loads and improved histopathology, with significant implications for health management in the aquaculture industry [14,15].

The pressing need to enhance both the nutritional value of macroalgae and increase aquaculture production sustainability has led researchers and practitioners to turn to these natural dietary supplements. These supplements are particularly targeted at improving the health and productivity of economically important aquaculture species, like the Asian seabass. This research paper aims to delve into the potential health benefits and operational enhancements that *U. rigida* extract supplementation could offer to Asian seabass aquaculture, focusing on growth performance, intestinal microbiota, antioxidant, immune responses, and disease resistance, thereby contributing to the sustainable intensification of aquaculture and supporting global seafood security.

## 2. Materials and Methods

### 2.1. Extraction, Phytochemical Compositions, and Antioxidant Activity of U. rigida

#### 2.1.1. Preparation and Extraction of *U. rigida*

The sea lettuce was harvested from earthen ponds in Phetchaburi Coastal Aquaculture Research and Development Center in Phetchaburi province, Thailand, in January 2023, where the seawater salinity was 20 ppt. After harvesting, the seaweed was washed with tap water to remove all mud and epiphytes, and then it was dried and blended. Subsequently, it was extracted using continuously boiled water at 90 °C for 1 h, with a seaweed to solvent (distilled water) ratio of 100 g per 500 mL. The *U. rigida* hot water crude extract (*Ur*-HWCE) was obtained by filtering from solid residues and then removing the solvent through freeze-drying over a period of 48 h. The resulting product, *Ur*-HWCE powder, was then stored at 4 °C for future experiments. The percentage yield (% yield) of the crude extract was calculated following = (weight of crude extract obtained)/(initial weight of dried seaweed used) × 100.

#### 2.1.2. Chemical and Structural Analyses of Sulfated Polysaccharides (SPs)

The sulfate content was determined according to the protocol described by [16]. The structural analysis of the active components in *Ur*-HWCE was conducted using Fourier-transform infrared (FTIR) spectroscopy with a PERKIN ELMER Spectrum One (Waltham, MA, USA). *Ur*-HWCE was combined with KBr to create a transparent film. Spectral analysis was performed over the frequency range of 4000 to 400 cm^−1^, with the vibration spectra graphically recorded.

#### 2.1.3. Nutritional Profiles and Fatty Acid Analysis

The nutritional profiles, including protein, fat, total carbohydrate, total protein, moisture, total energy, energy from fat, and fatty acid analysis, encompassing both saturated and unsaturated fatty acids, were measured by the Institute of Food Research and Product Development, Kasetsart University, Thailand, according to the protocol outlined by AOAC in 2023 [17].

#### 2.1.4. Total Phenolic Content (TPC)

Total phenolics in *Ur*-HWCE were determined using the Folin–Ciocalteu reagent, following the method described by Wolfe et al. (2003) [18] with slight modifications. A total of 0.1 mL of the extract (5 mg/mL) was mixed with 10% Folin–Ciocalteu reagent (Sigma-Aldrich, Singapore) and 2.0 mL of 7.5% sodium carbonate. After 20 min of incubation at RT, absorbance was measured at 760 nm using a spectrophotometer (GENESYS 20, Thermo Fisher Scientific, San Diego, CA, USA). The analysis was performed in triplicate, and the results were expressed as mg gallic acid equivalent per gram of extract (mg GAE/g extract).

#### 2.1.5. Total Flavonoid Content (TFC)

Total flavonoids in *Ur*-HWCE were determined using the method described by Ordonez et al. (2006) [19]. Briefly, 0.1 mL of the extract was mixed with 0.5 mL of 2% aluminum chloride (RCI Labscan, Bangkok, Thailand), incubated for 60 min, and measured for absorbance at 420 nm. The analysis was performed in triplicate, and the results were expressed as mg quercetin equivalent per gram of extract (mg QE/g extract).

#### 2.1.6. Total Tannins

Determination of tannins in *Ur*-HWCE was performed following the method of Tamilselvi et al. (2012) [20] he with a minor modification. In brief, 0.1 mL of *Ur*-HWCE was mixed with 5.0 mL of Folin–Ciocalteu reagent (7.5% *v*/*v*) (Loba Chemie, Maharashtra, India) and 1.0 mL of sodium carbonate solution (35% *w*/*v*) (Sigma-Aldrich, Singapore). The reaction mixture was mixed and incubated at room temperature for 30 min. The absorbance was measured at 725 nm. The analysis was performed in triplicate. The content of tannins in *Ur*-HWCE was expressed as milligrams of tannic acid equivalent per gram of an extract (mgTAE/g extract).

#### 2.1.7. Total Saponins

The content of saponins in *Ur*-HWCE was estimated using the method of Anhwange et al. (2004) [21] with a minor modification. *Ur*-HWCE (100 mg) was dissolved in 10 mL of aqueous ethanol (20% *v*/*v*), shaken vigorously, and heated at 55 °C for 90 min. The mixture was filtered through Whatman No. 1 filter paper. The residue was dissolved in aqueous ethanol and mixed with diethyl ether (VWR Chemicals BDH, Radnor, PA, USA). The aqueous layer was discarded. The saponins were shaken with *n*-butanol (RCI Labscan, Thailand) and washed with sodium chloride (5% *w*/*v*) (RCI Labscan, Bangkok, Thailand). The saponin extract was evaporated to dryness. The analysis was performed in triplicate. The saponin content was expressed as milligrams of saponin per gram of extract (mg/g extract).

#### 2.1.8. Total Antioxidant Assay (TAA)

The total antioxidant capacity of the *Ur*-HWCE was assessed using a method based on Umamaheswari and Chatterjee (2008) [22]. The extract (0.2 mL) was mixed with 2.0 mL of a reagent solution containing 0.6 M sulfuric acid (RCI Labscan, Bangkok, Thailand), 28 mM sodium phosphate (Sigma-Aldrich, Singapore), and 4 mM ammonium molybdate (Sigma-Aldrich, Singapore). After incubation at 95 °C for 90 min, the absorbance at 695 nm was measured in triplicate. The total antioxidant capacity was then calculated and expressed as milligrams of gallic acid equivalent per gram of extract (ugGAE//g extract).

#### 2.1.9. ABTS Radical Scavenging Activity

The 2,2′-azino-bis-(3-ethylbenzothiazoline-6-sulfonic acid) (ABTS) radical scavenging activity of the extract was determined following the method outlined by Re et al. (1999) [23]. ABTS radicals were generated by mixing 7.46 mM ABTS (Sigma-Aldrich, Singapore) with potassium persulfate (Loba Chemie, Maharashtra, India) and incubating overnight in darkness. The resulting ABTS solution was diluted with deionized water to an absorbance of 0.70 ± 0.02 at 734 nm. Various concentrations of the 0.01 mL of *Ur*-HWCE were added to 1.0 mL of the ABTS solution, and the decrease in absorbance from blue-green to clear color was measured after 1 min using a spectrophotometer (GENESYS 20, Thermo Fisher Scientific, CA, USA). The analysis was conducted in triplicate. The percentage inhibition was calculated using the following formula:

% inhibition = [A_initial_ − A_final_/A_initial_] × 100

A_initial_ and A_final_ were absorbances at 734 nm at 0 min and 1 min, respectively. The IC_50_ value, representing the concentration of the extract reducing 50% of the ABTS radicals, was calculated from the percentage inhibition data.

#### 2.1.10. DPPH Radical Scavenging Activity

The antioxidant activity of the *Ur*-HWCE in scavenging 2,2-diphenyl-1-picrylhydrazyl (DPPH) radicals was assessed using the method described by Susanti et al. (2007) [24]. Various concentrations of the extract (100 μL) were added to 2.0 mL of 0.13 mM methanolic DPPH (Sigma-Aldrich, Singapore) solution. The initial absorbance (A_initial_) was measured promptly at 517 nm. After incubating the mixture for 30 min at room temperature in darkness, the final absorbance (A_final_) was recorded. The analysis was conducted in triplicate. The percentage inhibition was calculated using the following formula:

% inhibition = [A_initial_ − A_final_/A_initial_] × 100

A_initial_ and A_final_ were absorbances at 517 nm at 0 min and 30 min. The percentage of inhibition was used to calculate the IC_50_ value.

#### 2.1.11. Total Iron Reducing Power Assay

The Fe^3+^ to Fe^2+^ reducing ability was determined using Gülçin’s method (2006) [25]. The *Ur*-HWCE (0.1 mL) at various concentrations was mixed with a phosphate buffer (200 mM, pH 6.6), potassium ferricyanide (10 mg/mL) (Sigma-Aldrich, Singapore), and incubated at 50 °C for 20 min. After adding trichloroacetic acid (10% *w*/*v*) (Sigma-Aldrich, Singapore) to stop the reaction, the mixture was incubated with ferric chloride (0.1% *w*/*v*) (Fluka, Darmstadt, Germany) and distilled water. The absorbance of the resulting green-chromogen was measured at 700 nm after a 10 min incubation to assess reducing ability.

#### 2.1.12. Anti-Lipid Peroxidation Assay

The anti-lipid peroxidation activity of *Ur*-HWCE was determined based on a modified method described by Lin and Yen (1999) [26]. Various concentrations of *Ur*-EWCE (1–200 mg/mL, 0.5 mL) were mixed with 0.5 mL of a phosphate buffer (pH 7.4), 1 mL of linoleic acid emulsion (Sigma-Aldrich, Singapore), 0.2 mL of 0.01% *w/v* FeSO_4_ (Sigma-Aldrich, Singapore), and 0.2 mL of 0.02% *w*/*v* ascorbic acid (Sigma-Aldrich, Singapore). The mixture was then incubated at 37 °C for 12 h. After incubation, the reaction mixture was combined with 0.2 mL of 4% *w*/*v* trichloroacetic acid (TCA) (Sigma-Aldrich, Singapore), 2 mL of 0.8% *w*/*v* thiobarbituric acid (TBA) (Sigma-Aldrich, Singapore), and 0.2 mL of 0.4% *w*/*v* butylated hydroxytoluene (BHT) (Loba Chemie, Maharashtra, India). The mixture was heated at 100 °C for 30 min. After cooling to room temperature, 2 mL of chloroform was added for extraction. The upper layer was collected, and its absorbance was measured at 532 nm. Deionized water was used as the blank. The percentage inhibition of lipid peroxidation by *Ur*-HWCE was calculated using the following formula:

% inhibition = [A_blank_ − A_sample_/A_blank_] × 100

where A_blank_ represents the absorbance of the blank and A_sample_ represents the absorbance of the *Ur*-HWCE. The percentage of inhibition was used to calculate the IC_50_ value.

#### 2.1.13. Nitric Oxide Scavenging Activity

The scavenging activity of *Ur*-HWCE against nitric oxide (NO) was determined based on a modified procedure described by Farhan et al. (2012) [27]. Various concentrations of *Ur*-EWCE (1–200 mg/mL, 0.25 mL) were mixed with a reaction mixture containing 10 mM sodium nitroprusside (1 mL) (Sigma-Aldrich, Singapore) and 0.5 M phosphate buffer solution (0.25 mL, pH 7.4). The mixture was then incubated at 25 °C for 30 min. After the incubation, 1 mL of Griess reagent (a mixture of 0.1% *N*-1-naphthyl-ethylenediamine dihydrochloride (Sigma-Aldrich, Singapore), 2% phosphoric acid (Sigma-Aldrich, Singapore), and 1% sulfanilamide (Sigma-Aldrich, Singapore)) was added. The pink chromogen formed from the diazotization of nitrite ions with sulfanilamide, followed by coupling with α-naphthyl-ethylenediamine, was measured at 540 mm using a spectrophotometer (GENESYS 20, Thermo Fisher Scientific, USA). The blank consisted of all the reactants, except the *Ur*-HWCE. The percentage of NO scavenging activity of *Ur*-HWCE was calculated using the following formula:

% inhibition = [A_blank_ − A_sample_/A_blank_] × 100

where A_blank_ represents the absorbance of the blank and A_sample_ represents the absorbance of the *Ur*-HWCE. The percentage of inhibition was used to calculate the IC_50_ value.

#### 2.1.14. Hydrogen Peroxide Scavenging Activity

The scavenging activity of *Ur*-HWCE against hydrogen peroxide (H_2_O_2_) was determined based on a modified protocol of Ruch et al. (1989) [28]. Various concentrations of *Ur*-EWCE (1–200 mg/mL, 0.5 mL) were mixed with 1 mL of 40 mM H_2_O_2_ (Sigma-Aldrich, Singapore) prepared in a phosphate buffer (pH 7.4). The mixture was incubated at room temperature for 10 min, after which the absorbance was measured at 230 nm. The phosphate buffer was used as the blank. The percentage of hydrogen peroxide scavenging activity of *Ur*-HWCE was calculated using the following formula:

% inhibition = [A_blank_ − A_sample_/A_blank_] × 100

where A_blank_ represents the absorbance of the blank and A_sample_ represents the absorbance of the *Ur*-HWCE. The percentage of inhibition was used to calculate the IC_50_ value.

#### 2.1.15. Hydroxyl Radical Scavenging Activity

The scavenging activity of *Ur*-HWCE against the hydroxyl radical was determined based on Fenton’s reaction, using Fe^3+^-EDTA-ascorbic acid and H_2_O_2_ reaction mixture, as described by Halliwell and Gutteridge (1984) and Harsha and Latha (2012) [29,30]. Various concentrations of *Ur*-EWCE (1–200 mg/mL, 100 μL) were mixed with a reaction mixture containing 100 μL of 28 mM 2-deoxy-2-ribose (Sigma-Aldrich, Singapore), 300 μL of 20 mM KH_2_PO_4_-KOH buffer (pH 7.4), 100 μL of 200 μM FeCl_3_ (Sigma-Aldrich, Singapore), 200 μL of 1.04 mM EDTA (Sigma-Aldrich, Singapore), 100 μL of 1 mM H_2_O_2_ (Sigma-Aldrich, Singapore), and 100 μL of 1 mM ascorbic acid (Sigma-Aldrich, Singapore). The mixture was incubated at 37 °C for 1 h. After incubation, 1 mL of 1% *w*/*v* TBA (Sigma-Aldrich, Singapore) and 1 mL of 2.8%*w*/*v* TCA (Sigma-Aldrich, Singapore) were added, followed by boiling at 100 °C for 20 min. The absorbance of the resulting pink chromogen was measured at 532 nm. The blank consisted of all the reactants, except *Ur*-HWCE. The percentage of hydroxyl radical scavenging activity was calculated using the following formula:

% inhibition = [A_blank_ − A_sample_/A_blank_] × 100

where A_blank_ represents the absorbance of the blank and A_sample_ represents the absorbance of the *Ur*-HWCE. The percentage of inhibition was used to calculate the IC_50_ value.

#### 2.1.16. Superoxide Radical Scavenging Activity

The scavenging activity of *Ur*-HWCE against superoxide radicals was determined using a modified method described by Hazra et al. (2008) [31]. Various concentrations of *Ur*-EWCE (1–200 mg/mL, 100 μL) were mixed with a reaction mixture consisting of 20 mM riboflavin (Sigma-Aldrich, Singapore), 12 mM EDTA (Sigma-Aldrich, Singapore), 50 mM sodium phosphate buffer (pH 7.6), and 0.1% *w*/*v* nitroblue tetrazolium (NBT) (Sigma-Aldrich, Singapore). The mixture was then illuminated with light for 90 s, after which the absorbance was measured at 562 nm. A phosphate buffer was used as the blank. The percentage of superoxide radical scavenging activity was calculated using the following formula:

% inhibition = [A_blank_ − A_sample_/A_blank_] × 100

where A_blank_ represents the absorbance of the blank and A_sample_ represents the absorbance of the *Ur*-HWCE. The percentage of inhibition was used to calculate the IC_50_ value.

### 2.2. Effects of Ur-HWCE Supplemented Feed on Health Benefits in Asian Seabass

#### 2.2.1. Ethics Statement

All experimental procedures involving aquatic animals strictly followed the Ethical Principles and Guidelines for the Use of Animals established by the National Research Council of Thailand for scientific purposes. Additionally, the protocol received approval from the Animal Ethics Committee at Kasetsart University, Thailand (Ethics ID: ACKU67-FIS-011), on 20 May 2024.

#### 2.2.2. Animal Husbandry

Healthy Asian seabass, initially weighing 2.0 ± 0.2 g, were purchased from a hatchery farm in Samut Sakhon province, Thailand. The fish were acclimated to laboratory conditions for fourteen days prior to the start of the experiment. During acclimation, they were kept in a 3000 L quarantine tank with continuous aeration. The temperature was maintained at 28 ± 2 °C, pH 7 ± 1, and a 12 h light/12 h dark photoperiod. Dissolved oxygen (DO) levels were kept between 5 and 7 mg/L. Salinity was maintained at 5 ppt throughout the acclimation and experimental periods. Fish were fed twice daily at 9:00 a.m. and 4:00 p.m., amounting to 5% of their body weight per feeding. Approximately 30% of the water in the tanks was changed daily to maintain optimal water quality for Asian seabass. Weekly, water samples from the rearing tanks were collected and analyzed for total ammonia, nitrite, alkalinity, and hardness following the APHA et al. (1995) method [32].

#### 2.2.3. Experimental Design

In this study, the experimental design included four groups, each assigned to randomized 250 L tanks following a completely randomized design (CRD). A total of 48 fish, with an average weight of approximately 5.0 ± 0.6 g following the acclimatization period, were allocated to each treatment group for a duration of 4 weeks. Each group included four replicates of 12 fish each. The control group received commercial feed mixed with sterile dH_2_O at 10% *w*/*w*, while the experimental groups were fed feed mixed with *Ur*-HWCE at three different concentrations: 0.5, 1.0, and 5 g/kg of feed for four weeks. The *Ur*-HWCE at different concentrations was dissolved in sterile distilled water at a ratio of 100 mL per 1 kg of feed and used to coat the surface of the feed pellets. The mixed feed was allowed to dry in a forced-air drier at room temperature for 30 min before being provided to the fish. Experimental feeds were freshly prepared daily for this study. The carnivorous basal feed composition consisted of 40% crude protein (Uni-President Ltd., Nakhon Pathom, Thailand), administered two times a day, equivalent to 5% of their body weight.

#### 2.2.4. Growth Performance Analysis

Fish growth performance was evaluated at the beginning and at the end of the 4-week experiment. Before measurements, all fish were anesthetized with a 10 ppm clove oil solution for 2 min to record their weight and note any mortalities. Weight gain (WG), average daily gain (ADG), specific growth rate (SGR), feed conversion ratio (FCR), and survival rate were calculated using the following equations [33]:

WG (g/fish) = final weight − initial weight

ADG (g/day) = (final weight − initial weight)/experimental period

SGR (%/day) = (ln (final body weight) − ln (initial body weight)/experimental period) × 100

FCR = feed intake (g)/total weight gain (g)

Survival rate (%) = (final total number of fish/initial total number of fish) × 100

#### 2.2.5. Serum and Peripheral Blood Leukocyte (PBL) Isolation

Non-anticoagulated blood was allowed to clot at room temperature for 1 h, after which the serum was separated by centrifugation at 3500× *g* for 15 min and stored at −20 °C for further analyses, including blood biochemistry, oxidative stress markers, antioxidant activity, and humoral immune responses. Leukocytes were isolated separately from whole blood following the method previously described by [34] for phagocytosis analysis. Blood was drawn from the caudal vein using a heparinized syringe and mixed with RPMI-1640 solution at a 1:2 ratio to prepare cell suspensions. These suspensions were layered onto Histopaque-1077, (Sigma-Aldrich, Singapore) density gradients at a 1:1 ratio and centrifuged at 800× *g* for 30 min. Cells at the gradient interface were collected and washed twice with PBS, pH 7.4. The concentration and viability of the leukocyte suspensions were assessed by counting under a microscope with trypan blue staining.

#### 2.2.6. qRT-PCR Analysis of Growth and Immune-Related Genes

At the end of the experiment, in week 4, the brain, liver, PBLs, head kidney, and intestine tissues were collected and preserved in 1.0 mL TRIzol™ reagent (Invitrogen, San Francisco, CA, USA) and shortly kept at −80 °C for further total RNA isolation assay. Total RNA extraction from these tissues was conducted following the manufacturer’s protocol. The concentration and purity of the extracted RNA were assessed using a NanoDropTM spectrophotometer (Thermo Fisher Scientific, CA, USA). To synthesize first-strand cDNA, 1 microliter of 1000 ng/μL total RNA was utilized as a template with Maxime™ RT PreMix (iNtRON Biotechnology, Seongnam-si, Republic of Korea). The resulting first-strand cDNA products were stored at −80 °C for subsequent gene expression analysis.

The synthesized cDNA from brain and liver tissues was used for evaluating growth-related gene expression, while cDNA from liver, whole blood, head kidney, and intestine tissues was analyzed for immune-related gene expression. The growth-related gene targeted was *igf1*, and immune-related genes included *dcs*, *c3*, *igm*, *lyz*, *il8*, and *il10.*

For qRT-PCR analysis, Brilliant III Ultra-Fast SYBR^®^ Green (Agilent, Santa Clara, CA, USA) was used in an AriaMx Real-Time instrument (Agilent, CA, USA). The qRT-PCR cycling conditions consisted of an initial denaturation step at 95 °C for 5 min, followed by 40 cycles of 95 °C for 30 s, 60 °C for 30 s, and 72 °C for 90 s, with a final extension at 72 °C for 10 min. The three housekeeping genes including *actb*, *gapdh,* and *ef1a* were used as an internal control to normalize mRNA and cDNA quantities and ensure result consistency. Relative expression of all target genes in fish tissues was determined using 2^−ΔΔCT^ analysis. All primers were validated and underwent efficacy testing prior to conducting the experiments. The sequences for all targeted genes are detailed in Table 1.

#### 2.2.7. Measurement of Oxidative Stress Biochemical Markers in Serum

**(1) Thiobarbituric acid reactive substances assay (TBARS assay)**

Malondialdehyde (MDA) levels in serum were determined using the TBARS assay [41]. Serum (0.1 mL) was mixed with normal saline solution (0.85% *w*/*v*), thiobarbituric acid (0.2 mL), and trichloroacetic acid (1.0 mL). The mixture was incubated at 100 °C for 30 min and cooled, and then 2.0 mL of distilled water was added. After centrifugation at 3500 rpm for 10 min, the supernatant was measured at 532 nm using a spectrophotometer against a blank. MDA concentration in the sample was determined using a tetramethoxypropane standard curve and expressed as μmole/mg protein.

**(2) Catalase (CAT)**

CAT activities were investigated using the modified method of Maehly (2006) [42]. The assay was carried out using a reaction mixture composed of 0.1 mL of serum, 2.5 mL of 50 mM phosphate buffer at pH 5.0, and 0.4 mL of 5.9 mM hydrogen peroxide. Absorbance readings were taken at 240 nm at 30 s intervals over a 2 min period. A standard curve was generated using a known catalase standard, and the catalase (CAT) activity in the sample was calculated and reported as units per minute per milligram of protein.

**(3) Superoxide dismutase (SOD)**

SOD activities were measured using the method of Takada et al. (1982) [43]. A 0.1 mL aliquot of serum was combined with 1.0 mL of a reaction solution containing 0.1 mM xanthine, 0.025 mM nitroblue tetrazolium (NBT), 0.1 mM disodium EDTA, 60 mM sodium carbonate buffer (pH 10.2), and xanthine oxidase. The absorbance was monitored at 560 nm to assess activity. A standard curve was prepared using known superoxide dismutase (SOD) concentrations, and enzyme activity was expressed as units per minute per milligram of protein.

**(4) Reduced glutathione (GSH)**

The content of GSH was determined using the modified method of Jollow et al. (1974) [44]. To prepare the reaction, 1.0 mL of 4% (*w*/*v*) sulfosalicylic acid was mixed with 1.0 mL of serum and incubated at 4 °C for 1 h. The mixture was then centrifuged at 3500 rpm for 20 min at 4 °C, and the resulting supernatant was collected. This supernatant was subsequently combined with a solution containing 100 mM phosphate buffer (pH 7.4) and 100 mM DTNB. The absorbance of the resulting yellow-colored compound was measured at 412 nm. A standard curve for reduced glutathione (GSH) was generated, and the GSH concentration in the sample was reported in nmoles per milligram of protein.

**(5) Glutathione peroxidase (GPx)**

GPx enzyme activity was measured following Mohandas et al. (1984) [45]. A total of 1.9 mL of a reaction solution containing a 0.1 M phosphate buffer (pH 7.4), 1.0 mM EDTA, 1.0 mM sodium azide, 1.0 U/mL glutathione reductase, 1.0 mM glutathione, 0.2 mM NADPH, and 0.25 mM hydrogen peroxide was combined with 0.1 mL of the serum sample. The activity of glutathione peroxidase (GPx) was assessed by measuring the reduction in NADPH absorbance at 340 nm at 25 °C. GPx activity was reported as micromoles of NADPH oxidized per minute per milligram of protein.

**(6) Glutathione S-transferase (GST)**

GST activity was measured according to Habig et al. (1974) [46]. A reaction mixture was prepared by combining 1.475 mL of a 0.1 M phosphate buffer (pH 6.5), 0.2 mL of 1.0 mM reduced glutathione, 0.025 mL of 1.0 mM 1-chloro-2,4-dinitrobenzene (CDNB), and 0.3 mL of serum. The formation of the CDNB–glutathione conjugate was monitored by measuring the absorbance at 340 nm. Glutathione S-transferase (GST) activity was calculated and reported as nanomoles of conjugate formed per minute per milligram of protein.

#### 2.2.8. Cellular and Humoral Immune Response Assays

**(1) Lysozyme activity**

Serum lysozyme activity was evaluated based on the lysis of *Micrococcus lysodeikticus* (Sigma, MO, USA), as previously described [36]. Lysozyme activity levels were determined using the following formula:

Enzyme (units/mL) = [(A_540_ sample − A_540_ blank) dilution factor]/(0.001) × (0.1)

**(2) Phagocytic activity**

Phagocytic activity was evaluated using a modified version of the previously described method [47]. Initially, 200 μL of PBLs at a concentration of 1 × 10^6^ cells/mL were loaded onto glass coverslips and allowed to adhere for 90 min. Following this, the coverslips were washed twice with RPMI medium before adding 200 μL of RPMI containing approximately 1 × 10^8^ particles/mL of latex beads (Sigma-Aldrich, USA). After incubating for 90 min, coverslips were washed twice to remove non-phagocytosed beads. The cells were then stained using a Dip-Quick staining kit (VR Bioscience, Thailand) and examined microscopically, counting 100 cells per slide. Phagocytic activity (PA) and the phagocytic index (PI) were calculated using the following formulas:

PA = (number of phagocytic cells/number of total cells count) × 100

PI = (number of total bead particles/number of total phagocytic cells) × 100.

**(3) Bactericidal activity**

Serum bactericidal activity against live *V. vulnificus* strain AAHM-VV2312 was assessed using serum collected from experimental fish. In each assay, 40 µL of serum was combined with 10 μL of 1 × 10^5^ CFU/mL bacterial suspension in 2.0% NaCl solution within a 96-well plate, resulting in a final bacterial count of 1 × 10^3^ CFU. The mixture was then incubated for 2 h at RT. Surviving bacteria were enumerated after 24 h of incubation on thiosulfate citrate bile salts sucrose (TCBS) agar plates. Negative controls (without serum) and positive controls (without bacteria) were included to determine 100% survival and 100% bactericidal activity, respectively. The bactericidal activity was calculated as the percentage of surviving bacteria after exposure to serum and plating for 24 h using the following formula:

BA (%) = [(T_0_ − T_24_)/T_0_] × 100,

where T_0_ is the number of initial bacteria and T_24_ is the number of bacteria after plating for 24 h.

**(4) Total serum IgM antibody level**

Serum IgM antibody levels were quantified by ELISA as previously described by [1,37,48] with some modifications. Initially, microplates were coated with a bicarbonate/carbonate coating buffer (pH 9.6) for 2 h at RT. After discarding the buffer, 100 μL of fish serum (diluted 1:100) was added to each well and incubated overnight at 4 °C. Following three washes with PBST (phosphate-buffered saline with 0.05% Tween-20, pH 7.4), the wells were blocked with 100 μL of VisualProtein-BlockPRO™ Blocking Buffer for 2 h at RT. After another round of washing, rabbit anti-Asian seabass IgM monoclonal antibody (GeneScript, NJ, USA) was added and incubated for 2 h at RT as in the previous study [49]. The plates were washed again and then incubated with horseradish peroxidase-conjugated anti-rabbit IgG (diluted 1:5000) for 2 h at RT. Following six washes, TMB One Component HRP Microwell Substrate was added (100 μL per well) and incubated at RT for 1 min in the dark. The reaction was stopped with 100 μL of TMB Stop Solution. The absorbance was measured at 450 nm using an iMark™ Microplate Absorbance Reader (Bio-Rad Laboratories Ltd., Hercules, CA, USA). Negative control wells without fish sera were used to establish the cutoff absorbance.

#### 2.2.9. Blood Biochemical Analysis

At the end of the experiment, serum samples from Section 2.2.5 were promptly processed to measure the following 23 biochemical parameters: albumin (ALB), total protein (TP), alkaline phosphatase (ALP), alanine aminotransferase (ALT), aspartate aminotransferase (AST), gamma-glutamyl transferase (GGT), direct bilirubin (DBIL), total bilirubin (TBIL), globulin (GLB), indirect bilirubin (IBIL), the albumin/globulin ratio (ALB/GLB), calcium (Ca), creatinine (Crea), total carbon dioxide (tCO2), phosphorus (P), cholesterol (CHOL), glucose (GLU), urea, amylase (AMY), creatine kinase (CK), the urea/creatinine ratio (Urea/Crea), total bile acids (TBAs), and the AST/ALT ratio (AST/ALT). The biochemical analyses were performed using the MSC100V veterinary coagulation and chemistry combo analyzer (Zhejiang PushKang Biotechnology Co., Ltd., Shaoxing, Zhejiang, China), with reagent kits supplied by the same manufacturer.

#### 2.2.10. Gut Microbiota Analysis

A mid-intestine section (approximately 2 cm in length) was randomly collected from three fish per group and homogenized for bacterial DNA extraction using the ZymoBIOMICS™ DNA Miniprep Kit (Zymo Research, Irvine, CA, USA). Sequencing and analysis were performed by Biomarker Technologies Co., Ltd. (Münster, Germany). The full-length 16S rRNA gene was sequenced using the PacBio platform. Marker genes were sequenced using the SMRT Cell method, followed by circular consensus sequencing (CCS) filtering for OTU clustering, taxonomic annotation, and abundance analysis. Specific barcoded primers based on 16S full-length primers 27F (5′-AGRGTTTGATYNTGGCTCAG-3′) and 1492R (5′-TASGGHTACCTTGTTASGACTT-3′) were used for PCR amplification. The purified and quantified products were homogenized to form a sequencing library (SMRT Bell) and sequenced by PacBio Sequel.

The raw sequencing data were processed using QIIME2 (version 2023.2; https://qiime2.org, accessed on 2 August 2023). Taxonomic classification was performed using a pretrained Naive Bayes classifier based on the SILVA 138 reference database (https://www.arb-silva.de/documentation/release-138/, accessed on 10 November 2023). Data normalization was conducted using the Total Sum Scaling (TSS) method. Subsequently, alpha diversity (within-sample diversity) and beta diversity (between-sample diversity) indices were calculated to assess microbial community structure and variation [50].

#### 2.2.11. Histological Analysis

At the end of the trial, the liver, head kidney, and intestine (continuous with the part used for gut microbiota analysis) were collected and preserved in 10% formalin. The tissues were embedded in paraffin and sectioned into 3–5 µm thickness using a microtome. The hematoxylin and eosin (H&E) staining process involved removing paraffin from tissue sections with xylene, rehydrating them through graded alcohols to distilled water, staining nuclei with Harris’ hematoxylin, washing, differentiating with 1% acid alcohol, neutralizing with 0.2% ammonia water, counterstaining the cytoplasm with eosin, dehydrating through graded alcohols, and clearing with xylene.

The Periodic acid–Schiff (PAS) staining process involved deparaffinizing and hydrating tissue slides, oxidizing in 0.5% periodic acid for 5 min, staining with Schiff reagent for 15 min, rinsing in lukewarm water, counterstaining with hematoxylin for 1 min, and then dehydrating, clearing, and mounting. Glycogen and mucins appeared magenta, while nuclei stained blue.

The Alcian blue (pH 2.5) staining process involved deparaffinizing and hydrating tissue slides, staining with Alcian blue for 45 min, rinsing, counterstaining with nuclear fast red for 5 min, and then dehydrating, clearing, and mounting. Acidic mucins stained blue, while nuclei and cytoplasm stained pink to red. All slides were finally mounted with Permount™ solution for microscopic examination (Olympus, Westborough, MA, USA) to evaluate histopathological alterations.

#### 2.2.12. Disease Resistance and Relative Percent Survival (RPS) to *Vibrio vulnificus*

After the feeding trial, 40 fish per group (10 fish per replicate) were randomly selected and housed individually in 250 L fiberglass tanks. Each fish received an intraperitoneal injection of 100 μL containing 1 × 10^6^ CFU/mL *V. vulnificus* AAHM-VV2312 (1 × 10^5^ CFU/fish), determined from the bacteria’s LD_50_ preliminary test on the fish. Daily mortality rates were recorded for 14 days, and the relative percent survival (RPS) value was calculated as follows: RPS = 1 − (mortality rate of treatment fish/mortality rate of control fish) × 100 [51].

#### 2.2.13. Statistical and Data Analysis

Growth performance, immune-related gene expressions, antioxidant activity, cumulative survival, biochemical parameters, and RPS are expressed as mean ± standard deviation (SD). Data were statistically analyzed by one-way analysis of variance (ANOVA). Duncan’s multiple range test (DMRT) was used to assess significant differences among treatment groups. All statistical analyses were performed using Statistical Package for Social Science (SPSS for Mac version 24.0 Chicago, IL, USA). The cumulative survival analysis of fish challenged with *V. vulnificus* AAHM-VV2312 was calculated using the Kaplan–Meier method. Cumulative survival plots were generated using SPSS for Mac (version 24.0, Chicago, IL, USA). Statistical significance between the control and treatment groups was denoted by letter superscripts and * at *p* < 0.05.

## 3. Results

### 3.1. Proximate Composition, Chemical and Phytochemical Contents, and Antioxidant Activity of Ur-HWCE

A total yield of 28.33% dry crude extract was obtained from dried seaweed extracted with hot water (Ur-HWCE). The analysis of Ur-HWCE revealed a proximate composition consisting of 6.75% total protein, 57.63% total carbohydrate, 0.26% fat, 31.96% ash, 3.40% moisture, and 6.01% sulfate polysaccharide. The fatty acid profile included 0.01% lauric acid (C12:0), 0.14% palmitic acid (C16:0), 0.04% stearic acid (C18:0), 0.04% oleic acid (C18:1n9c), and 0.01% linoleic acid (C18:2n6C). The bioactive compounds present were measured as 2.33 ± 0.28 mg GAE/mg extract for total phenolics, 2.41 ± 0.25 mg QE/mg extract for total flavonoids, and 11.86 ± 1.76 µg GAE/g extract for total antioxidants. The total tannin content was 1.05 ± 0.18 mg TAE/g extract. Additionally, the total saponin content was measured at 0.37 ± 0.06 mg/g extract.

Additionally, the antioxidant activities demonstrated IC_50_ values of 18.23 ± 0.32 mg/mL for ABTS radical scavenging, 112.24 ± 10.75 mg/mL for DPPH radical scavenging, and an EC_50_ of 108.33 ± 13.29 mg/mL for reducing power. Furthermore, the extract exhibited notable inhibitory effects against various oxidative stress markers. The IC_50_ values were 92.7 ± 2.1 mg/mL for anti-lipid peroxidation activity, 88.5 ± 1.6 mg/mL for nitric oxide scavenging activity, 91.2 ± 1.8 mg/mL for hydrogen peroxide scavenging activity, 85.6 ± 2.0 mg/mL for hydroxyl radical scavenging activity, and 89.8 ± 1.9 mg/mL for superoxide radical scavenging activity (Table 2).

### 3.2. Structural Analyses of Crude Polysaccharides from Ur-HWCE

**Figure 1:**
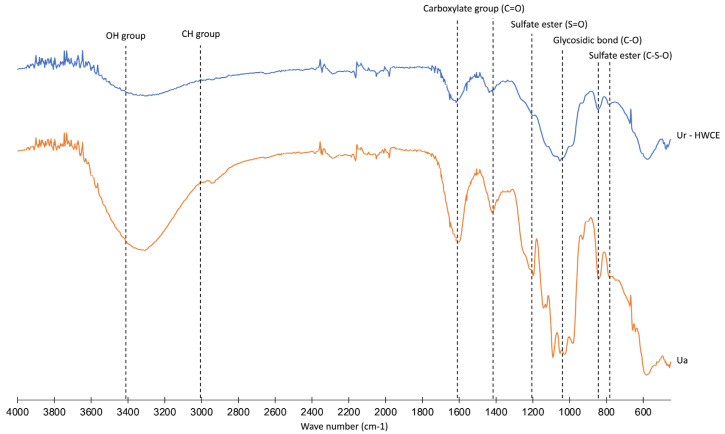
The structure of *Ur*-HWCE analyzed by Fourier-transform infrared (FTIR) spectroscopy compared to the commercial ulvan from *U. amoricana* (*Ua*).

The FTIR spectrum analysis of the *Ur*-HWCE sample from the seaweed extract revealed a rich composition of biochemical molecules, as evidenced by distinct absorption peaks. Notably, the broad absorption around 3400 cm^−1^ indicated hydroxyl (OH) groups, typical of polysaccharides and phenolic compounds, suggesting hydration properties or hydrogen bonding potential. The presence of aliphatic CH groups was marked by peaks between 3000 cm^−1^, likely reflecting fatty acids or lipid structures. Carboxylate groups were clearly identified by the sharp peak at approximately 1600 cm^−1^, indicative of carboxylic acids or esters. Additionally, the spectrum showed specific peaks for sulfate esters around 1200 cm^−1^ and below 1000 cm^−1^, characteristic of sulfated polysaccharides, like fucoidans, which are prevalent in seaweeds. Glycosidic bonds, confirmed by peaks near 1000 cm^−1^, reinforced the presence of polysaccharides (Figure 1).

### 3.3. Effects of Ur-HWCE Dietary Supplement on Health Benefits in Asian Seabass

#### 3.3.1. Growth Performance and Survival

Throughout the experiment, the water conditions remained stable and conducive to the health and growth of the Asian seabass, ensuring suitability for fish rearing. Dissolved oxygen levels consistently exceeded 5 mg/L, pH ranged from 6.5 to 8.5, and temperatures were maintained between 25 and 28 °C. Total ammonia concentrations ranged from 1.0 to 1.2 mg/L, alkalinity varied from 50 to 300 mg/L as CaCO_3_, and total hardness was within the range of 150–300 mg/L and the salinity of 5 ppt.

The growth performance of fish fed varying concentrations of Ur-HWCE in their diet showed consistent results across all groups with non-significant differences (*p* > 0.05), with weight gain ranging from 30.13 g to 32.38 g, average daily gain from 1.00 g to 1.08 g, specific growth rate from 4.04% to 4.90%, and the feed conversion ratio from 1.48 to 1.60. Survival rates were consistently at 100% across all groups (Table 3).

#### 3.3.2. Growth-Related Gene Expression

**Figure 2:**
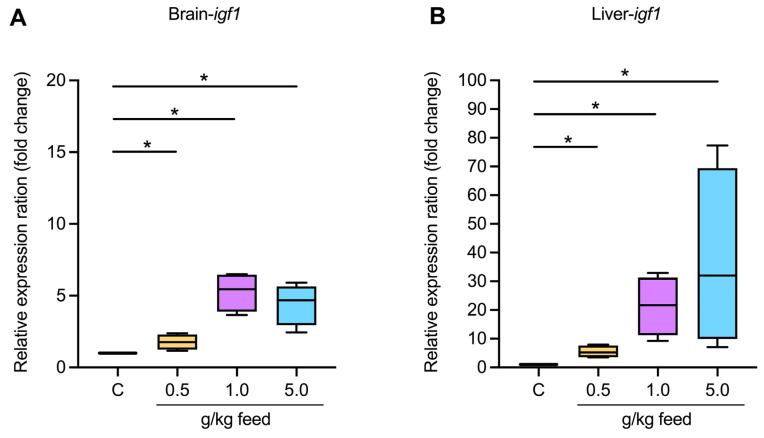
The expression of growth-related gene *igf1* in the brain (**A**) and liver (**B**) of Asian seabass feeding with *Ur*-HWCE supplementation for 4 weeks. The * on the graph indicates the presence of statistically significant differences among groups at a significance level of *p* < 0.05.

The relative expression of insulin-like growth factor 1 (*igf1*) in both the brain and liver was examined across various feed concentrations (0.5 g/kg, 1.0 g/kg, and 5.0 g/kg), showing significant upregulation compared to a control group (*p* < 0.05) **(**Figure 2A**)**. In the brain, *igf1* expression increased between 1.76- and 5.26-fold, with the highest increases observed at 1.0 g/kg. Similarly, liver *igf1* expression exhibited a noticeable dose-dependent increase, ranging from 5.47- to 37.11-fold, with the most significant expression at 5.0 g/kg (Figure 2B). These results highlight a strong positive correlation between increased feed concentration and *igf1* expression in both the brain and liver, indicating potential benefits for metabolic and growth processes within these tissues.

#### 3.3.3. Oxidative Stress Biochemical Markers in Serum

**Figure 3:**
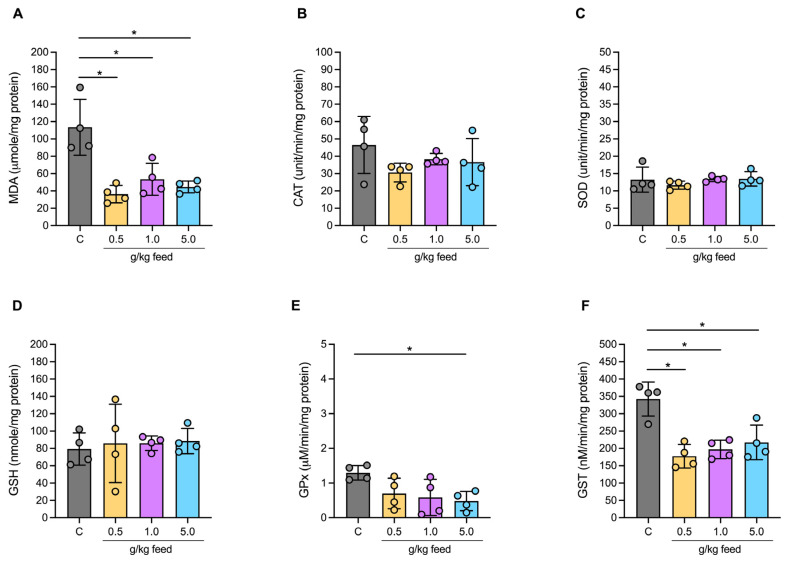
Antioxidative markers and enzymatic activity in serum of MDA (**A**), CAT (**B**), SOD (**C**), GSH (**D**), GPx (**E**), and GST (**F**) of Asian seabass feeding with *Ur*-HWCE supplement for 4 weeks (*n* = 4). The * on the graph indicates the presence of statistically significant differences among groups at a significance level of *p* < 0.05.

The MDA levels, indicative of lipid peroxidation in fish serum, decreased significantly in all tested seaweed extract dosages (0, 0.5, 1.0, and 5.0 g/kg feed), with values ranging from 36.43 to 53.46 μmole/mg protein (*p* < 0.05) (Figure 3A). The activity of glutathione peroxidase (GPx) also decreased significantly with increasing seaweed extract dosage, particularly at 5.0 g/kg, where it reached 0.48 μmole/mg protein (*p* < 0.05) (Figure 3E). Additionally, GST activity showed a noticeable decrease across all tested dosages of seaweed extract, with activity levels ranging from 177.5 to 217.3 nM/min/mg protein, which were significantly lower compared to the control group (*p* < 0.05) (Figure 3F).

There were no significant differences in CAT, SOD, and GSH activities among all treatment groups when compared to the control group (*p* > 0.05) (Figure 3B–D).

#### 3.3.4. Humoral and Cellular Immune Responses

**Figure 4:**
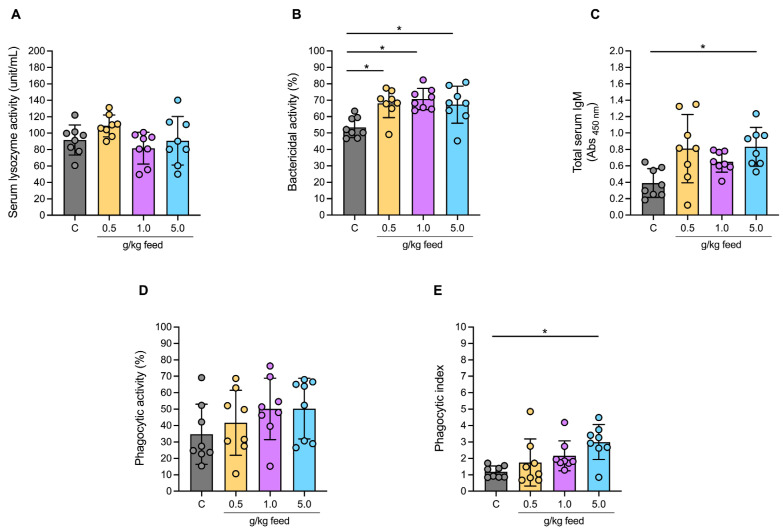
Humoral and cellular immune responses of serum lysozyme (**A**), bactericidal activity (**B**), total serum IgM (**C**), phagocytic activity (**D**), and phagocytic index (**E**) of Asian seabass feeding with *Ur*-HWCE supplement for 4 weeks (*n* = 8). The * on the graph indicates the presence of statistically significant differences among groups at a significance level of *p* < 0.05.

Serum lysozyme activity was not statistically significant among the control group and all treatment groups (*p* > 0.05) (Figure 4A). Meanwhile, bactericidal activity demonstrated a significant increase across all feed concentration groups (0.5 g/kg, 1.0 g/kg, and 5.0 g/kg), ranging from 67.29% to 70.30% compared to the control group, which showed the lowest activity at 53.48% (*p* < 0.05) (Figure 4B). Total serum IgM levels markedly increased at the highest feed concentration of 5.0 g/kg, with a significant difference compared to the control group, which had the lowest IgM levels. Lower concentrations (0.5 g/kg and 1.0 g/kg) tended to show higher increases but did not exhibit significant changes compared to the control group (*p* > 0.05) (Figure 4C). Phagocytic activity revealed a clear dose-dependent increase with higher feed concentrations but did not reach statistical significance. The control group displayed the lowest activity, while phagocytic activity progressively rose with increasing feed levels, reaching the highest at 5.0 g/kg (Figure 4D). The phagocytic index showed a significant increase at the highest feed concentration of 5.0 g/kg compared to the control group, which had the lowest index. Lower concentrations (0.5 g/kg and 1.0 g/kg) did not show significant changes but tended to increase the phagocytic index (*p* > 0.05) (Figure 4E).

#### 3.3.5. Immune-Related Gene Expression

**Figure 5:**
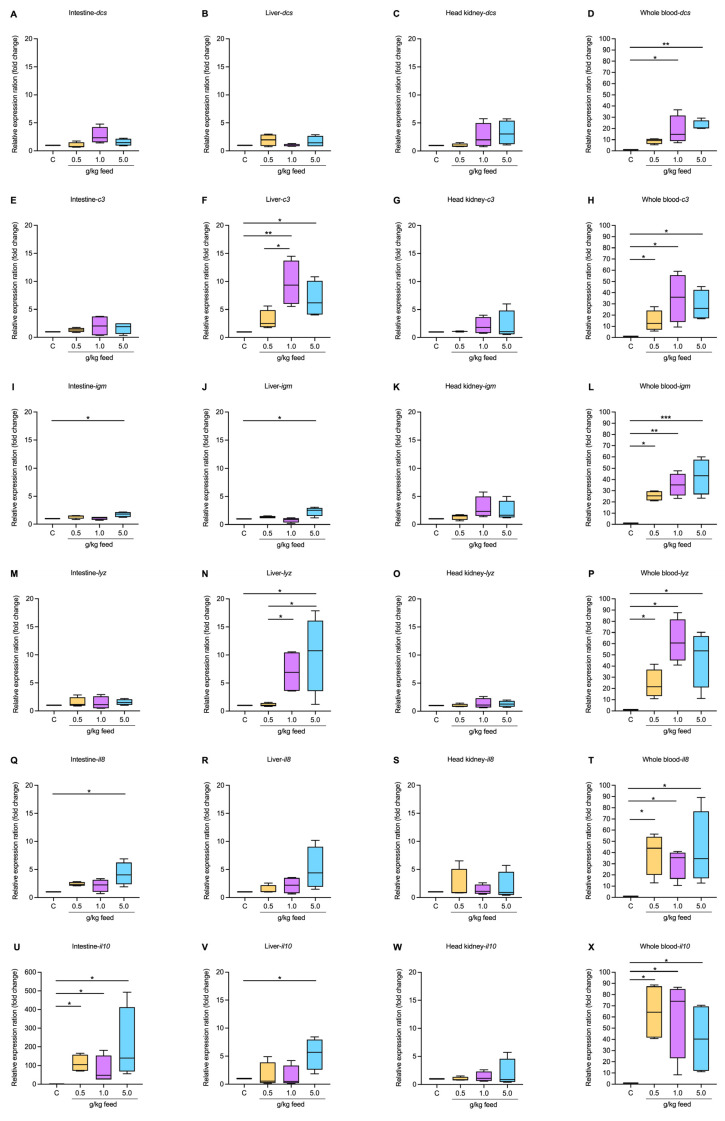
The expression of immune-related genes in the intestine, liver, head kidney, and whole blood of Asian seabass feeding with *Ur*-HWCE supplement for 4 weeks including *dcs* (**A**–**D**), *c3* (**E**–**H**), *igm* (**I**–**L**), *lyz* (**M**–**P**), *il8* (**Q**–**T**), and *il10* (**U**–**X**). The *, **, and *** on the graph indicate the presence of statistically significant differences among groups at a significance level of *p* < 0.05, *p* < 0.01, and *p* < 0.001, respectively.

The study on the impact of seaweed extract on dendritic cell-specific (*dcs*) gene expression in various tissues of fish demonstrated that dietary supplementation with seaweed extract, at the tested dosages (control, 0.5, 1.0, 5.0 g/kg feed), did not significantly alter the expression in the intestine, liver, or head kidney. However, a notable significant upregulation was observed in whole blood, where the expression significantly increased at the 1.0 and 5.0 g/kg groups, which were higher by 18.39- and 22.64-fold changes, respectively, when compared to the control group (*p* < 0.05 and *p* < 0.01, respectively) (Figure 5A–D).

The complement component 3 (*c3*) in the intestine showed a moderate increase at higher doses, while in the liver, *c3* expression significantly increased, particularly at 1.0 and 5.0 g/kg. The head kidney displayed stable *c3* expression with only a subtle increase at the highest dosage. In whole blood, there was a significant increase in *c3* expression with increasing seaweed extract dosage, especially noted at the higher concentrations of 1.0 and 5.0 g/kg, which ranged from 14.56- to 35.15-fold compared to the control group (*p* < 0.05) (Figure 5E–H).

Similar trends are observed in the expression of immunoglobulin M (*ighm*), lysozymes (*lyz*), interleukin-8 (*il8*), and interleukin-10 (*il10*) across various tissues, with similar patterns indicating enhanced expression, particularly at higher seaweed extract dosages. Notably, the expression levels of *ighm* were significantly upregulated at 5.0 g/kg in the intestine, liver, and whole blood, with strong observations also made at 0.5 and 1.0 g/kg of whole blood, which exhibited higher significant differences (*p* < 0.05). There were no significant differences in *ighm* expression in the head kidney, but the trend was higher at higher doses (*p* > 0.05) (Figure 5I–L).

The *lyz* gene expression was significantly upregulated in the liver and whole blood across all treated concentrations compared to the control group (*p* < 0.05). However, there were no significant differences in lysozyme expression in the intestine and head kidney (*p* > 0.05) (Figure 5M–P).

The *il8* expression showed substantial increases in upregulation expression in the intestine at the highest dosage of 5.0 g/kg, as well as across all tested dosages in whole blood when compared to the control group (*p* < 0.05). However, there were no significant differences in *il8* expression in the liver and head kidney (*p* > 0.05) (Figure 5Q–T). All tested dosages of seaweed extract in the intestine and whole blood resulted in a substantial upregulation of *il10* expression, with the highest dosage of 5.0 g/kg showing significant increases in the liver. However, there were no significant differences in *il10* expression in the head kidney (*p* > 0.05) (Figure 5U–X).

#### 3.3.6. Blood Biochemical Analysis

**Figure 6:**
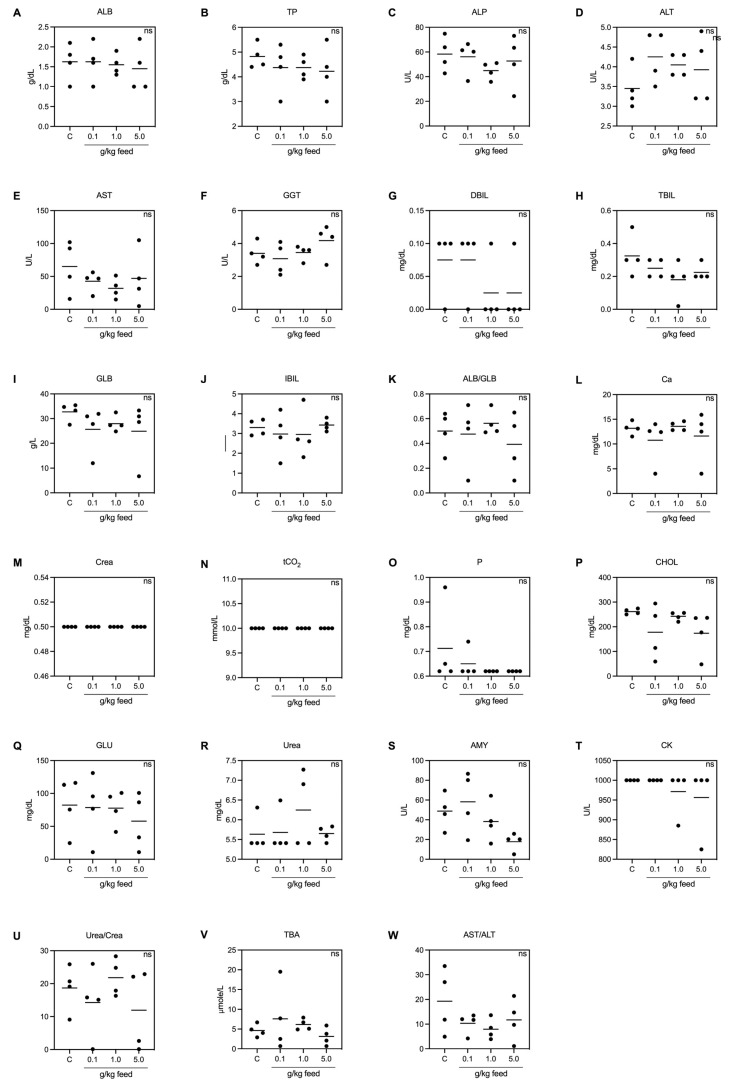
Blood biochemical profile of Asian seabass feeding with *Ur*-HWCE supplement for 4 weeks (*n* = 4) including ALB, albumin (**A**); TP, total protein (**B**); ALP, alkaline phosphatase (**C**); ALT, alanine aminotransferase (**D**); AST, aspartate aminotransferase (**E**); GGT, gamma-glutamyl transferase (**F**); DBIL, direct bilirubin (**G**); TBIL, total bilirubin (**H**); GLB, globulin (**I**); IBIL, indirect bilirubin (**J**); ALB/GLB, albumin/globulin ratio (**K**); Ca, calcium (**L**); Crea, creatinine (**M**); tCO_2_, total carbon dioxide (**N**); P, phosphorus (**O**); CHOL, cholesterol (**P**); GLU, glucose (**Q**); Urea (**R**); AMY, amylase (**S**); CK, creatine kinase (**T**); Urea/Crea, urea/creatinine ratio (**U**); TBAs, total bile acids (**V**); AST/ALT, aspartate transaminase/alanine transaminase ratio (**W**); ns: not significant differences.

The biochemical parameters measured across various dosages of seaweed extract suggest that the supplement did not adversely affect liver, kidney, bone, or metabolic functions in fish, supporting its safe inclusion in their diet at the tested concentrations. The tests indicated no significant differences in all tested blood biochemical parameters, including levels of albumin, alkaline phosphatase, alanine aminotransferase, aspartate aminotransferase, gamma-glutamyl transferase, direct bilirubin, total bilirubin, globulin, calcium, creatinine, total carbon dioxide, phosphorus, cholesterol, glucose, urea, amylase, creatine kinase, the urea/creatinine ratio, total bile acids, and the AST/ALT ratio (Figure 6A–W).

#### 3.3.7. Gut Microbiota Analysis

**Figure 7:**
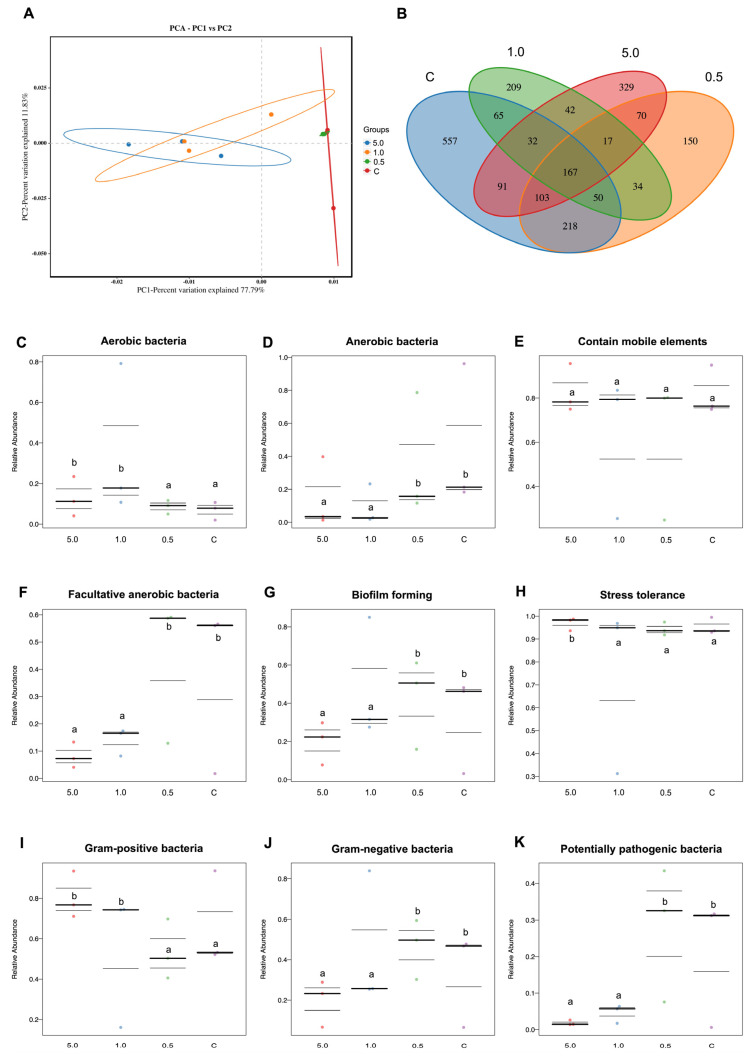
The PCA plot (**A**) and the Venn diagram (**B**) of the 16s RNA intestinal microbiota sequencing samples (*n* = 4). It represents the average relative abundance of the respective functional group within the samples including aerobic bacteria (**C**), anaerobic bacteria (**D**), contains mobile elements (**E**), facultatively anaerobic bacteria (**F**), biofilm forming (**G**), stress-tolerant bacteria (**H**), Gram-positive bacteria (**I**), Gram-negative bacteria (**J**), and potentially pathogenic bacteria (**K**). Differing letters indicate statistically significant differences between groups at a significance level of *p* < 0.05.

The PCA plot clearly classified the samples into distinct groups corresponding to the two main groups: one with the control and 0.5 g/kg feed and the other with 1.0 g/kg feed and 5.0 g/kg feed (Figure 7A). Similarly, the Venn diagram effectively illustrated the differentiation and overlapping areas among the sample groups. This diagram highlighted the unique bacterial populations, showing counts of 557, 150, 209, and 329 common microbial counts in the control, 0.5 g/kg feed, 1.0 g/kg feed, and 5.0 g/kg feed groups, respectively (Figure 7B).

The relative abundance of aerobic bacteria showed a decreasing trend from 5.0 to the control groups (*p* > 0.05) (Figure 7C). Meanwhile, anaerobic bacteria exhibited an increasing relative abundance from 5.0 to the control groups (*p* < 0.05) (Figure 7D). There were no significant differences in the prevalence of bacteria containing mobile elements among all treatment groups (*p* > 0.05) (Figure 7E). The facultative anaerobic bacteria maintained a relatively higher abundance across different groups, with the highest increase observed at 0.5 g/kg feed and the control groups (*p* < 0.05) (Figure 7F). Biofilm-forming bacteria showed a higher relative abundance in the 0.5 g/kg feed and control groups compared to 1.0 and 5.0 g/kg feed (*p* < 0.05) (Figure 7G). The number of stress-tolerant bacteria exhibited high relative abundance in 5.0 g/kg feed compared to the remaining groups of 1.0 and 0.5 g/kg feed and the control groups (*p* < 0.05) (Figure 7H). There was a gradual increase in the relative abundance of Gram-positive bacteria from the group of 1.0 and 5.0 g/kg feed compared to the 0.5 g/kg feed and control groups (Figure 7I). Meanwhile, Gram-negative bacteria also showed an increased abundance within the 0.5 g/kg feed and control groups, and it was lower in 1.0 and 5.0 g/kg feed (Figure 7J). All bacterial populations found in the intestines of all treatments exhibited that the abundance of potentially pathogenic bacteria was relatively low in 1.0 and 5.0 g/kg feed compared to the 0.5 g/kg feed and control groups, which had higher potentially pathogenic bacteria (Figure 7K).

**Figure 8:**
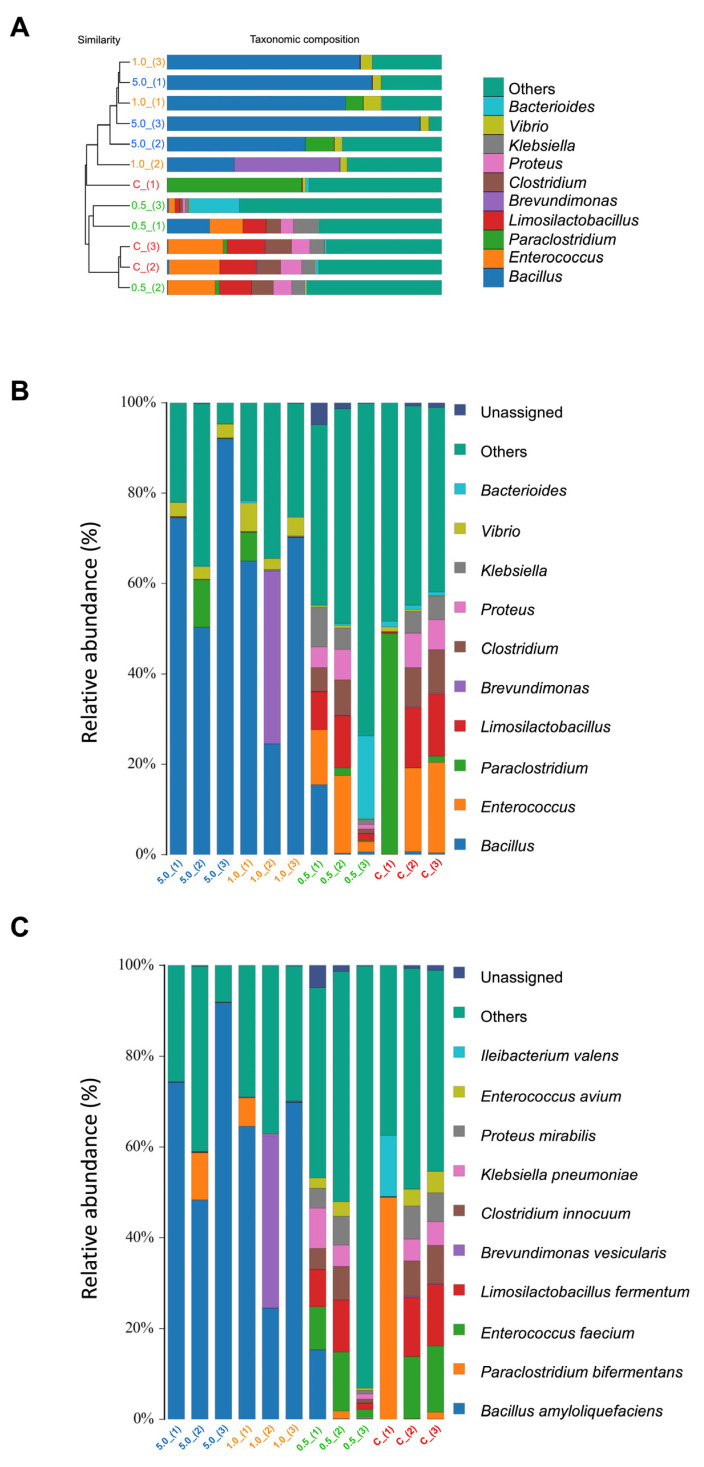
The phylogenetic taxonomic composition (**A**) and the relative abundance of various microbial functional groups in different treatments at the genus (**B**) and species (**C**) levels of Asian seabass feeding with *Ur*-HWCE supplement for 4 weeks (*n* = 4).

The analysis of the microbial communities across different concentrations (0.5, 1.0, and 5.0 g/kg feed) revealed a diverse range of bacteria prevalent in the samples. The taxa identified included genera such as *Vibrio*, *Klebsiella*, *Proteus*, *Clostridium*, *Brevundimonas*, *Limosilactobacillus*, *Paraclostridium*, *Enterococcus*, and *Bacillus* (Figure 8A).

The concentrations of microbial communities varied with the different groups. At higher concentrations (1.0 and 5.0 g/kg feed), there appeared to be a greater diversity of beneficial microbes, especially *Bacillus* spp. Meanwhile, between 0.5 g/kg feed and control, a similarity pattern with a lower presence of *Bacillus* spp. and a greater prevalence of *Bacteroides*, *Vibrio*, *Klebsiella*, *Proteus*, *Clostridium*, *Brevundimonas*, *Limosilactobacillus*, *Paraclostridium*, and *Enterococcus* was observed (Figure 8B).

In addition, the higher concentration of the *Ur*-HWCE, especially at 1.0 and 5.0 g/kg feed, could alter the gut microbiota by increasing the population of *Bacillus amyloliquefaciens*, enhancing the presence of beneficial microbes that might contribute to the stability and health of the microbial ecosystem compared to the 0.5 g/kg feed and control groups (Figure 8C).

#### 3.3.8. Histological Analysis

The histopathological examination of the liver, head kidney, and intestine in fish treated with *Ur*-HWCE showed that the organs maintained their typical structures. No severe abnormalities indicative of inflammation, such as immune cell infiltration or blunting and loss of villi, were observed in all tissues of the control and *Ur*-HWCE groups.

**Figure 9:**
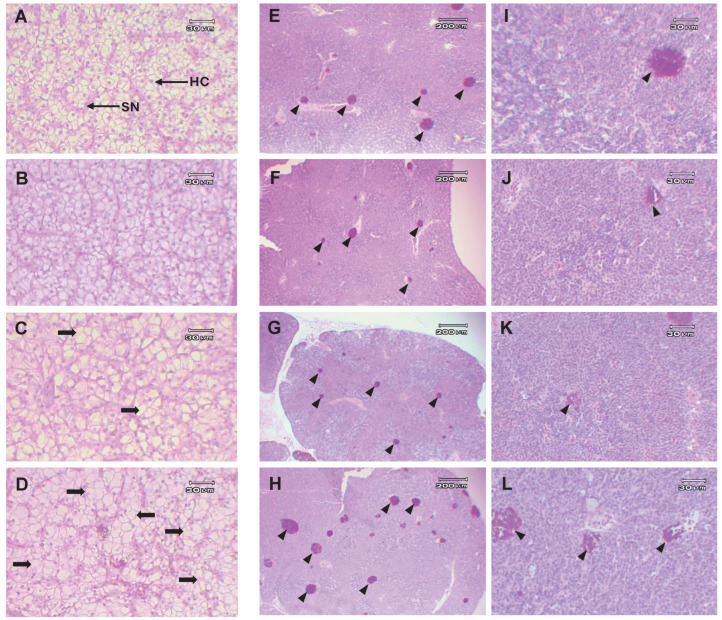
The histological examination of the liver (**A**–**D**) and head kidney (**E**–**L**) of Asian seabass fed with *Ur*-HWCE supplement for 4 weeks. Different concentrations of the *Ur*-HWCE supplement were tested: 0.5 g/kg feed (**B**,**F**,**J**), 1.0 g/kg feed (**C**,**G**,**K**), and 5.0 g/kg feed (**D**,**H**,**L**) and compared with the control group (**A**,**E**,**I**). H&E staining, HC, and hepatocytes with centrally located nuclei and abundant cytoplasm; SN, hepatic sinusoid; black arrow, slight vacuolization in the hepatocytes of the liver tissue; black arrow heads, melanomacrophage centers (MCCs).

The liver displayed a well-organized structure with distinct lobules separated by connective tissue septa. These hepatocytes, with centrally located nuclei and abundant cytoplasm, showed a polygonal shape in fish fed with 0.5 g/kg of *Ur*-HWCE. However, fish given 1.0 and 5.0 g/kg of *Ur*-HWCE exhibited slight vacuolization in the hepatocytes of the liver tissue (Figure 9A–D). Upon histopathological evaluation, the head kidney shows a slight increase in the number of melanomacrophage centers (MMCs), indicated by the small MCC in Figure 9H of the 5.0 g/kg of *Ur*-HWCE group compared to the control group and the 0.5 and 1.0 g/kg of *Ur*-HWCE. There are no severe histopathological alterations in the head kidney among all treatment groups. The intestinal tissues showed no differences between the control group and the 0.5, 1.0, and 5.0 g/kg of the *Ur*-HWCE treatment groups (Figure 9E–L).

**Figure 10:**
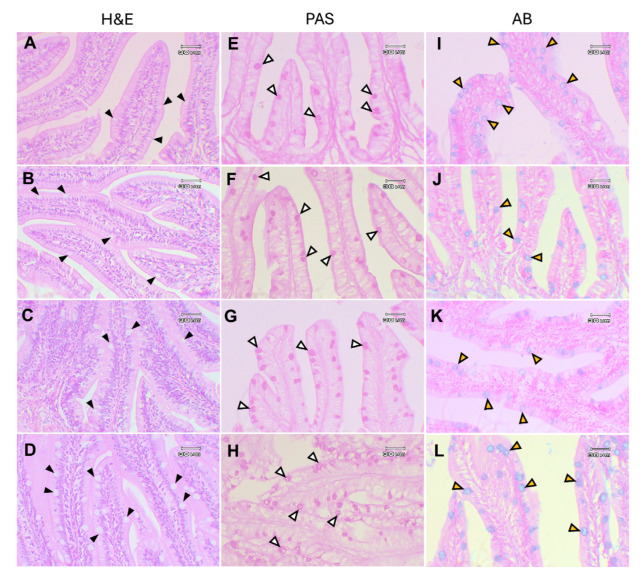
The intestinal histological examination of Asian seabass fed with *Ur*-HWCE supplement for 4 weeks stained with H&E (**A**–**D**), Periodic acid–Schiff (PAS) for neutral mucins (**E**–**H**), and Alcian blue (AB) for acidic mucins (**I**–**L**). There were no differences between the control group (**A**,**E**,**I**) and the 0.5 (**B**,**F**,**J**), 1.0 (**C**,**G**,**K**), and 5.0 (**D**,**H**,**L**) g/kg of *Ur*-HWCE treatment groups. Black head arrows, goblet cells; white head arrows, neutral mucins (stained pink); yellow head arrows, acidic mucins (stained blue).

The general structure of the small intestine tissue in Asian seabass showed no abnormalities in intestinal epithelial and goblet cells in any experimental groups (Figure 10A–D). Histochemical studies using PAS staining for neutral mucins (stained pink) (Figure 10E–H) and AB staining for acidic mucins (stained blue) in the intestine also showed no differences between the experimental groups (Figure 10I–L).

#### 3.3.9. Survival Rate and Relative Percent Survival (RPS) to *V. vulnificus* Challenge

**Figure 11:**
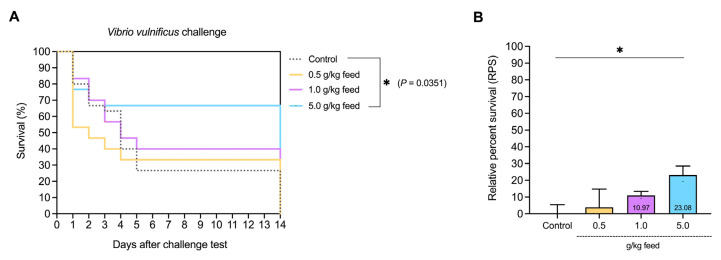
The survival plot (**A**) and relative percent survival (RPS) (**B**) of Asian seabass fed with *Ur*-HWCE supplement for 4 weeks and then challenged with virulent *Vibrio vulnificus* AAHM-VV2312. * indicates statistically significant differences between the treatment and control groups at a significance level of *p* < 0.05.

The percent survival over a 14-day period after a *Vibrio vulnificus* AAHM-VV2312 challenge showed a clear differentiation between the control group and the groups fed with varying concentrations of feed (0.5 g/kg, 1.0 g/kg, and 5.0 g/kg). The control group exhibited the lowest survival rate, while survival rates significantly increased in the feed groups. The highest survival rate of 66.66% was observed in the 5.0 g/kg feed group, compared to the control group, which had 26.66%, indicating that higher feed concentrations improved resistance to *Vibrio vulnificus* AAHM-VV2312 compared to the control (*p* = 0.0351) (Figure 11A). The relative percent survival (RPS) in response to different feed concentrations (0.5 g/kg, 1.0 g/kg, and 5.0 g/kg) showed a marked increase in RPS with higher feed concentrations compared to the control group, with the highest RPS observed at a 5.0 g/kg feed concentration of 23.08% (*p* < 0.05) (Figure 11B).

## 4. Discussion

The proximate analysis of *Ur*-HWCE showed high carbohydrate (57.63%), ash (31.96%), and moderate protein (6.75%) levels, along with sulfate polysaccharides (6.01%), indicating its potential bioactive and health-promoting properties in aquaculture species [52,53]. The detected fatty acids, though in minor quantities, are consistent with the lipid profiles found in other seaweed extracts and contribute to the overall nutritional value of the supplement [54,55]. FTIR analysis of *Ur*-HWCE confirmed the presence of polysaccharides, phenolics, and other bioactive compounds. Identified functional groups such as hydroxyl, aliphatic CH, carboxylate, sulfate esters, and glycosidic bonds correspond to ulvan, a known bioactive polysaccharide in sea lettuce [11]. This molecular complexity indicates potential for enhancing the nutritional value and health benefits of aquaculture feeds, as supported by studies showing that polysaccharide-rich seaweed extracts improve fish health and growth performance [56]. Phytochemical analysis revealed high levels of phenolics and flavonoids, contributing to the strong antioxidant activity of *Ur*-HWCE, as shown by ABTS, DPPH, and reducing power assays. These antioxidant capacities are comparable to other seaweed extracts, highlighting its potential as a functional feed additive to reduce oxidative stress in aquaculture [56,57].

Dietary supplementation with *Ur*-HWCE from *Ulva rigida* showed promising improvements in Asian seabass health without adverse effects. Growth performance indicators such as weight gain, average daily gain, specific growth rate, feed conversion ratio, and survival rate remained stable across all concentrations, consistent with previous studies where seaweed supplements supported high survival without compromising growth [58]. In addition, the significant upregulation of *igf1* expression in the brain and liver at higher *Ur*-HWCE concentrations suggests an enhancement of growth processes, aligning with findings where *igf1* expression correlated with improved growth and metabolic rates in fish [59]. The dose-dependent response suggests that *Ur*-HWCE may promote growth through endocrine modulation. *igf1* is a key regulator of growth and development, linked to increased cell proliferation and differentiation, which contributes to improved growth performance in fish [59,60]. *Ur*-HWCE may enhance growth by modulating the GH/IGF axis, stimulating GH secretion, and increasing *igf1* levels in target tissues. This promotes protein synthesis and cell proliferation, leading to improved growth performance in fish [61,62]. Prolonged feeding with *Ur*-HWCE may enhance growth performance through sustained activation of growth-promoting pathways. Continuous exposure to its bioactive compounds could lead to cumulative benefits over time. However, further research is needed to determine the long-term effects and optimal dosing strategies for its use in aquaculture.

The marked reduction in MDA activity with higher *Ur*-HWCE doses suggests strengthened antioxidative defense. This aligns with prior studies showing seaweed extracts lower oxidative stress, helping protect cellular integrity and support fish health [54]. By reducing oxidative stress, *Ur*-HWCE may improve overall fish health and metabolic efficiency, creating conditions that favor growth. Its antioxidant effects could also support endocrine function, enhancing the regulation of growth-related processes [63,64].

The increased bactericidal activity and elevated IgM levels at higher *Ur*-HWCE concentrations highlight its ability to enhance humoral immunity, aligning with studies showing the immune-boosting effects of seaweed-derived supplements in fish [65]. The increased phagocytic activity and index reinforce the immunostimulatory effects of *Ur*-HWCE, indicating enhanced pathogen resistance. This response likely involves multiple pathways, particularly humoral immunity. *Ur*-HWCE appears to stimulate the production of total IgM, the main antibody in early immune responses, which aids in pathogen neutralization and opsonization, enhancing phagocyte-mediated clearance [66]. The increased bactericidal activity suggests that *Ur*-HWCE may activate the complement system, leading to MAC formation and bacterial cell lysis, thereby strengthening immune defense [67]. In terms of cellular immunity, the increased phagocytic activity and index suggest that *Ur*-HWCE enhances macrophage and antigen-presenting cell function, which are key for pathogen clearance and initiating adaptive responses. It may also stimulate cytokine production, such as interleukins and interferons, which are vital for immune cell activation and regulation [68]. These mechanisms align with studies showing seaweed extracts boost fish immunity. Further research is needed to clarify the molecular pathways behind *Ur*-HWCE’s effects.

The unchanged lysozyme levels suggest that *Ur*-HWCE may have limited or delayed effects on this parameter. It is possible that longer exposure or higher dosages are needed to enhance lysozyme activity. The upregulation of immune-related genes (*dcs*, *c3*, *ighm*, *lyz*, *il8*, *il10*), especially at higher *Ur*-HWCE doses, suggests a broad immunomodulatory effect. This aligns with previous findings where seaweed polysaccharides enhanced immune gene expression in fish. Increased expression of genes like *dcs*, *c3*, and *lyz* indicates the activation of innate immunity, strengthening the fish’s rapid defense against infection [69,70]. Similarly, the upregulation of *ighm* indicates enhanced humoral immunity, essential for pathogen neutralization. Elevated *il8* and *il10* expressions reflect improved immune regulation, with *il8* recruiting immune cells to infection sites and *il10* controlling inflammation to prevent immune overactivation [71]. These findings highlight *Ur*-HWCE’s potential as a strong immunostimulant, enhancing disease resistance and overall fish health. Further research should investigate the specific immune pathways involved.

Gut microbiota analysis showed distinct shifts with increasing *Ur*-HWCE concentrations, particularly at 1.0 and 5.0 g/kg. These included higher levels of aerobic bacteria and *Bacillus* spp., notably *Bacillus amyloliquefaciens*, a known probiotic that supports gut health through antimicrobial production, improved nutrient absorption, and immune modulation [72,73]. Higher *Ur*-HWCE levels reduced potential pathogens and improved gut health, while lower doses favored biofilm formation and pathogen presence. The extract may create anaerobic conditions that inhibit biofilms and promote beneficial, stress-tolerant Gram-positive bacteria. Further research is needed to understand the mechanisms and optimize their use in aquaculture. Stable blood parameters across *Ur*-HWCE doses confirm its safety, aligning with studies showing no harm to fish organ function from seaweed supplements [64,74]. The lack of histopathological changes in the liver and intestine at high *Ur*-HWCE doses confirms its safety, consistent with studies showing no tissue damage from seaweed extracts [74].

Higher survival and RPS in *Ur*-HWCE-fed fish highlight its potential to boost resistance to *V. vulnificus*, consistent with studies showing that seaweed extracts enhance fish survival and immunity [14,70]. The increased survival rates likely result from *Ur*-HWCE’s immunostimulatory effects, enhancing both humoral and cellular responses. Upregulated immune genes, elevated IgM, improved phagocytic activity, and stronger bactericidal properties suggest better pathogen defense. These findings highlight *Ur*-HWCE’s potential as a functional supplement to improve fish health and disease resistance, supporting more sustainable aquaculture. Further studies are needed to clarify its mechanisms and optimize its application.

## 5. Conclusions

This study shows that *Ur*-HWCE from sea lettuce enhances growth, immunity, antioxidant defense, gut health, and disease resistance in Asian seabass without adverse effects. These results support its use as a functional feed additive in aquaculture. Further research should explore long-term effects and optimal dosing across species.

## Figures and Tables

**Table 1:** Primers used for gene expression analysis in this study.

Genes	Gene group	Primer Names	Nucleotide Sequences (5′→3′)	T_m_ (°C)	Reference
*Insulin-like growth factors (igf1)*	Growth-related gene	*Lc_Igf1*	F: ACGCTGCAGTTTGTATGTGG R: CCTTAGTCTTGGGAGGTGCA	60	XM_018697285.1
*Dendritic cells (dcs)*	Immune-related gene	*Lc_Dcs*	F: AAGACAGTAGACCTCTCCCACA R: CAAACAGGGGAAGGACTGAGAG	60	[35]
*Complement C3 (c3)*		*Lc_C3*	F: CATCACCAAAGAAATGCTGCCA R: CTCATAAGACGGAGCAGGTCTC	60	[36]
*Immunoglobulin m (ighm)*		*Lc_IgM*	F: TGTCAAGGTAAACGAGGGAGC R: TCCCCTGGATCCATTCGTCA	60	[37]
*Lysozyme (lyz)*		*Lc_Lyz*	F: TGCATCACACACCATGGCAA R: CATCCACGTTGTCATAGGAG	60	[36]
*Interleukin-8 (il8)*		*Lc_IL8*	F: GCATCATCAAGGAGAGAAAGCC R: GTGTCTGCTCAGCTTGTTTCTT	60	[38]
*Interleukin-10 (il10)*		*Lc_IL10*	F: GCTAGATCAGACCGTCGAAGAC R: TGACATCACTCTTGAGCTCGTC	60	[36]
*Actin beta (actb)*	References/housekeeping gene	*Lc_B-actin*	F: TACCACCGGTATCGTCATGGA R: CCACGCTCTGTCAGGATCTTC	60	[39]
*Elongation factor 1-alpha (ef1a)*		*Lc_ef1a*	F: GTTGCCTTTGTCCCCATCTC R: CTTCCAGCAGTGTGGTTCCA	60	[40]
*Glyceraldehyde 3-phosphate dehydrogenase (gapdh)*		*Lc_gapdh*	F: CGCTTCCTGCACAACCAACT R: GTGGCAGTGATGGCATGAAC	60	[40]

**Table 2:** Proximate composition, fatty acid profiles, bioactive compounds, and antioxidant properties of *Ur*-HWCE.

Analysis Parameters	Values
**Proximate composition**	
Total protein (%)	6.75
Total carbohydrate (%)	57.63
Fat (%)	0.26
Ash (%)	31.96
Moisture (%)	3.40
Sulfate polysaccharide (%)	6.01
**Fatty acids (%)**	
Saturated fatty acid: Lauric acid (C12:0)	0.01
Saturated fatty acid: Palmitic acid (C16:0)	0.14
Unsaturated fatty acid: Stearic acid (C18:0)	0.04
Unsaturated fatty acid: Oleic acid (C18:1n9c)	0.04
Linoleic acid (C18:2n6C)	0.01
**Bioactive compounds**	
Total phenolic content (mg GAE/g extract)	2.33 ± 0.28
Total flavonoid content (mg QE/g extract)	2.41 ± 0.25
Total tannins (mg TAE/g extract)	1.05 ± 0.18
Total saponins (mg/g extract)	0.37 ± 0.06
**Antioxidant activities**	
Total antioxidants (µg GAE/g extract)	11.86 ± 1.76
ABTS radical scavenging (IC_50_, mg/mL)	18.23 ± 0.32
DPPH radical scavenging (IC_50_, mg/mL)	112.24 ± 10.75
Reducing power (EC_50_, mg/mL)	108.33 ± 13.29
Anti-lipid peroxidation (IC_50_, mg/mL)	92.7 ± 2.1
Nitric oxide scavenging activity (IC_50_, mg/mL)	88.5 ± 1.6
Hydrogen peroxide scavenging activity (IC_50_, mg/mL)	91.2 ± 1.8
Hydroxyl radical scavenging activity (IC_50_, mg/mL)	85.6 ± 2.0
Superoxide radical scavenging activity (IC_50_, mg/mL)	89.8 ± 1.9

GAE: gallic acid equivalent, QE: quercetin equivalent.

**Table 3:** Growth performances and survival rates of Asian seabass feeding with *Ur*-HWCE supplementation for 4 weeks.

Groups	WG (g)	ADG (g)	SGR (%)	FCR	Survival Rate (%)
Control	31.38 ± 2.30	1.05 ± 0.08	4.04 ± 0.20	1.54 ± 0.09	100%
0.5 g/kg feed	32.38 ± 1.59	1.08 ± 0.05	4.90 ± 0.50	1.48 ± 0.07	100%
1.0 g/kg feed	30.13 ± 1.94	1.00 ± 0.06	4.71 ± 0.53	1.60 ± 0.10	100%
5.0 g/kg feed	31.5 ± 3.18	1.05 ± 0.11	4.82 ± 0.63	1.53 ± 0.15	100%

## Data Availability

Data are contained within the article.
